# Individualized DTI-ALPS Identifies Phase-Specific Glymphatic Dysfunction in Early-Stage Bipolar Disorder

**DOI:** 10.3390/biomedicines14030699

**Published:** 2026-03-17

**Authors:** Xiaoxi Zhao, Mingli Li, Qiang Wang, Lihong Deng, Liansheng Zhao, Hua Yu, Xiaojing Li, Wei Deng, Wanjun Guo, Tao Li, Wei Wei

**Affiliations:** 1 Affiliated Mental Health Center & Hangzhou Seventh People’s Hospital, Zhejiang University School of Medicine, Hangzhou 310013, China; 2 Nanhu Brain–Computer Interface Institute, Hangzhou 311121, China; 3 Mental Health Center of West China Hospital, Sichuan University, Chengdu 610041, China; 4 NHC and CAMS Key Laboratory of Medical Neurobiology, Zhejiang University, Hangzhou 310058, China; 5 Liangzhu Laboratory, MOE Frontier Science Center for Brain Science and Brain-Machine Integration, State Key Laboratory of Brain-Machine Intelligence, Zhejiang University, Hangzhou 311121, China; 6 Zhejiang Key Laboratory of Clinical and Basic Research for Psychiatric Diseases, Hangzhou 310013, China

**Keywords:** glymphatic system, DTI-ALPS, bipolar disorder, individualized ALPS pipeline

## Abstract

**Background**: The glymphatic system, essential for brain waste clearance and neuroimmune regulation, remains underexplored in the context of bipolar disorder (BD) among young populations. **Methods**: Using diffusion tensor image analysis along the perivascular space (DTI-ALPS), we compared ALPS indices derived from the conventional FSL-based (cFSL) pipeline with those from the individualized ALPS (iALPS) pipeline. A cohort of young adults comprising 77 individuals with BD and 289 healthy controls was analyzed to evaluate methodological consistency and to identify disorder-specific alterations in glymphatic function. **Results**: The two pipelines showed only moderate agreement (Lin’s concordance correlation coefficient = 0.52–0.60), suggesting that differences in ROI placement strategies significantly affect ALPS estimation. While the cFSL pipeline detected no group differences, the iALPS pipeline identified a trend-level reduction in ALPS index in patients with BD during depressive episodes, particularly in the right hemisphere (*p* = 0.036, uncorrected, FDR-adjusted *p* = 0.071). No significant glymphatic alterations were observed in individuals with early-stage BD. **Conclusions**: These findings suggest that glymphatic dysfunction in psychiatric disorders may be phase-specific on illness. The use of individualized and automated analytical strategies, such as the iALPS pipeline, appears to enhance sensitivity to subtle, state-related brain changes that conventional methods may overlook. This methodological advancement provides a more biologically informed framework for future large-scale and longitudinal studies aimed at elucidating the role of glymphatic function in the pathophysiology of psychiatric disorders.

## 1. Introduction

The discovery of the glymphatic system in the central nervous system (CNS) by Iliff et al. in 2012 introduced a new framework for understanding waste clearance in the brain [1] and has captured growing attention in both neuroscience and psychiatry research [2,3,4,5,6]. The glymphatic system is a macroscopic perivascular network that facilitates exchange between cerebrospinal fluid (CSF) and interstitial fluid (ISF), thereby promoting the clearance of metabolic waste from the CNS [7,8,9]. Anatomically, the glymphatic system comprises perivascular spaces (PVSs), CSF circulation pathways, and aquaporin-4 (AQP-4) water channels expressed on astrocytic endfeet. Within this system, CSF enters the brain parenchyma along arterial PVSs, exchanges with ISF via AQP4-mediated flux, and generates convective bulk flow that ultimately drains through venous PVSs, thereby facilitating the clearance of metabolic waste products [8,10,11]. Proper glymphatic activity is essential for neuroimmune and metabolic homeostasis, whereas its dysfunction may lead to the accumulation of metabolites and inflammatory factors, potentially triggering immunogenic and neurotoxic injury within the CNS [12,13,14].

Converging evidence suggests that neuroimmune dysregulation plays a pivotal role in the pathogenesis of major psychiatric disorders, including bipolar disorder (BD) [15,16,17]. Elevated levels of inflammatory cytokines and complement components have been consistently reported in BD [18,19]. Such immune disturbances can disrupt neurotransmission, induce aberrant synaptic pruning, and impair blood–brain barrier integrity [20,21,22]. Given the glymphatic system’s role in metabolite and immune factor clearance, its dysfunction might represent a shared neurobiological mechanism linking neuroimmune abnormalities with brain dysfunction in BD [23].

Recent advances in neuroimaging techniques have enabled non-invasive assessment of glymphatic function. In 2017, Taoka et al. proposed diffusion tensor image analysis along the PVS (DTI-ALPS) as a quantitative marker of glymphatic activity, based on direction-specific water diffusion along the PVSs [24]. The ALPS index leverages the distinct diffusion properties of water molecules in projection and association fiber regions adjacent to the lateral ventricles, where white matter tracts run approximately perpendicular to penetrating small vessels [25]. This orientation accentuates water diffusion within PVSs while minimizing confounding effects from the white matter’s intrinsic structure. Owing to its non-invasive and contrast-agent-free nature, the ALPS index has been applied to assess glymphatic function in various neurological conditions, including Alzheimer’s disease [26], Parkinson’s disease [27], and chronic insomnia [28]. Psychiatric researchers employing DTI-ALPS have mainly focused on schizophrenia (SCZ) [3,6] or major depressive disorders (MDDs) [29,30], often in chronic or older cohorts, lacking evidence specific to BD, particularly among young adults in different disease stages.

The original ALPS index calculation requires manual selection of regions of interest (ROIs) [24]. To eliminate the influence of manual selection of ROIs, conventional ALPS index calculation typically employs fixed, predefined ROI coordinates with standard space registration pipelines (e.g., DSI Studio or FSL). This fixed-coordinate approach neglects individual neuroanatomical variability, thereby limiting biological accuracy and reproducibility [25,30]. An individualized ALPS pipeline has recently been proposed to improve biological specificity by accounting for interindividual neuroanatomical variability during ROI selection (https://github.com/Winniework/Automated-DTI-ALPS-pipeline, accessed on 17 February 2025.) [31,32]. However, its performance and consistency relative to the conventional pipeline have not been systematically evaluated in psychiatric populations.

To address these gaps, the present study employed both the conventional FSL-based (cFSL) pipeline and the iALPS to evaluate glymphatic function among young adult cohorts with BD. By directly comparing the two analytical approaches, we aimed to (1) clarify whether glymphatic dysfunction is altered in patients with BD; and (2) evaluate the methodological reliability and specificity of the iALPS pipeline relative to the cFSL pipeline. Together, these efforts will help establish a more robust framework for quantitative glymphatic imaging and advance our understanding of the glymphatic system in the context of BD pathophysiology.

## 2. Materials and Methods

### 2.1. Participants

From October 2014 to June 2018, we recruited a total of 366 participants, including 289 healthy controls (HCs), 32 patients with bipolar disorder during a manic episode (BD-M), and 45 patients with bipolar disorder during a depressive episode (BD-D) from West China Hospital of Sichuan University. Demographic information, including age, sex, and years of education, was collected from all participants. Medication information for all patients is provided in Supplementary Table S1. All participants were Han Chinese and right-handed, as assessed by the Annett Handedness Scale [33]. All patients were interviewed with the Structured Clinical Interview for DSM-IV Disorders, Patient Version (SCID-4-P), and all met the DSM-IV diagnostic criteria for BD. Healthy controls were screened with the Structured Clinical Interview for DSM-IV Disorders, Non-patient Version (SCID-4-NP). Exclusion criteria comprised organic brain disorders, neurological diseases, severe endocrine or metabolic conditions, a history of alcohol or substance use disorders, intellectual disability, or a Wechsler Intelligence Scale score ≤ 70. Written informed consent was obtained from every participant. The research protocol was approved by the Ethics Committee of West China Hospital, Sichuan University.

### 2.2. Clinical and Cognitive Assessment

All patients underwent comprehensive clinical and cognitive assessments. Clinical symptoms were evaluated using standardized rating scales: Hamilton Depression Rating Scale (HAMD) and Young Mania Rating Scale (YMRS). Participants’ cognitive function was assessed using the abbreviated version of the Chinese Wechsler Adult Intelligence Scale [34].

### 2.3. MRI Data Acquisition

All participants were scanned using a Philips 3.0 T MRI system (Achieva; Philips, Amsterdam, The Netherlands). High-resolution T1-weighted (T1w) structural images and diffusion tensor imaging (DTI) data were acquired. During scanning, participants lay supine with eyes closed and wore earplugs to minimize scanner noise. Foam padding was used as needed to reduce head motion and prevent related artifacts. T1w structural images were acquired using a magnetization-prepared rapid gradient-echo (MPRAGE) sequence with the following parameters: TR = 2500 ms; TE = 8.1 ms; TI = 1072.4 ms; flip angle = 7°; slice thickness = 1 mm (no gap); voxel size = 1 × 1 × 1 mm^3^. DTI images were acquired by an echo-planer image (EPI) sequence with 2 diffusion gradient directions, b-values 0 and 1000, TR: 10,407 ms, TE: 92 ms, flip angle: 90°, FOV: 256 × 256 mm^2^, reconstructed voxel size 2 × 2 × 2 mm^3^, EPI factor 67, slice thickness: 2.0 mm, SENSE factor 2 in the anterior-posterior direction, 75 slices throughout the whole brain, strong fat suppression.

### 2.4. DTI-ALPS Index Calculation

#### 2.4.1. cFSL Pipeline

All raw diffusion-weighted imaging (DWI) data were preprocessed using the standard diffusion pipeline implemented in FSL (FMRIB Software Library, version 6.0). Head motion and eddy current-induced distortions were corrected using the eddy tool in FSL. The preprocessed diffusion data were fitted to a voxelwise diffusion tensor model using FSL’s ‘dtifit’ command. Subsequently, each subject’s fractional anisotropy (FA) map was registered to the ICBM FA template using the FLIRT tool [35]. Direction-specific diffusivity maps (Dxx, Dyy, Dzz) were extracted from the native space.

**Figure 1:**
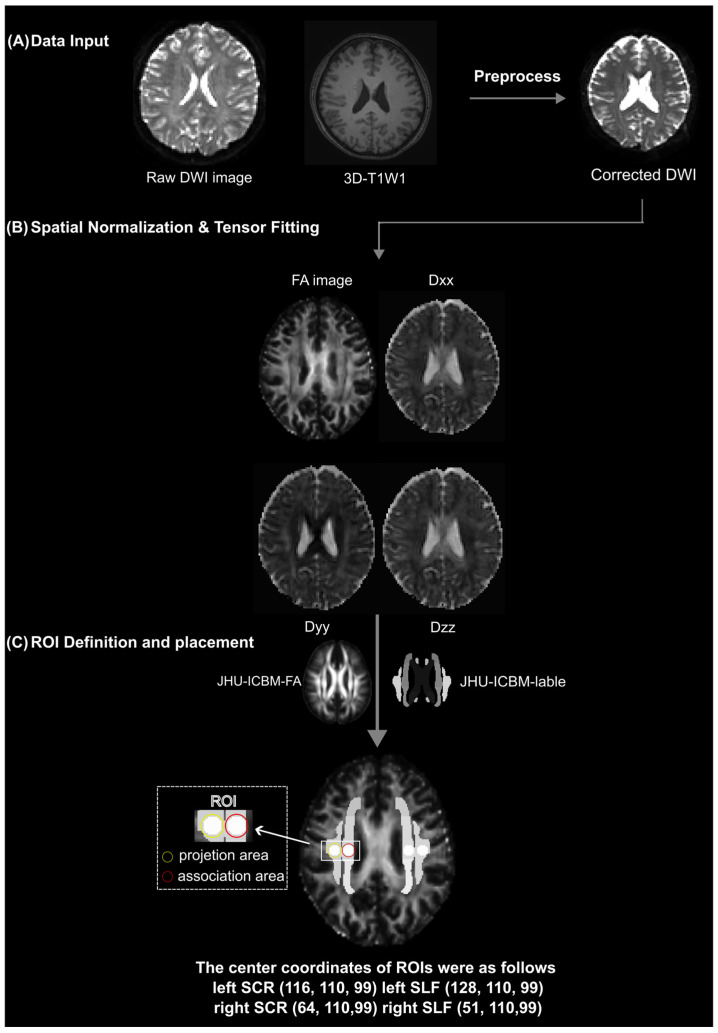
Workflow of the conventional FSL pipeline (cFSL) for DTI-ALPS index computation. The pipeline consists of three main steps. (**A**) Data input and preprocessing: raw diffusion-weighted imaging (DWI) and T1-weighted images were preprocessed using the standard FSL diffusion pipeline. The resulting artifact-corrected diffusion data were then used for further analysis. (**B**) Spatial normalization and tensor fitting: diffusion tensors were fitted at each voxel using FSL’s dtifit tool to generate FA, Dxx, Dyy, and Dzz maps. (**C**) ROI definition and placement: Each subject’s FA map was linearly registered to the ICBM FA template in MNI space, and the resulting transformation matrix was applied to the diffusivity maps later using vector-based registration (vecreg) to preserve tensor orientation. Spherical ROIs (radius = 2.5 mm) were programmatically defined at standardized coordinates within projection and association fiber tracts, as specified by the JHU-ICBM-labels-1 mm atlas. These standardized ROIs were inversely transformed into each subject’s native diffusion space using the previously computed transformation matrix, with nearest-neighbor interpolation to preserve mask integrity. All ROI placements were visually inspected, and no manual correction was required. DWI, diffusion-weighted imaging; T1w, T1-weighted; FA, fractional anisotropy; ROI, region of interest; MNI, Montreal Neurological Institute.

ROIs for DTI-ALPS index computation were defined in standard MNI space. Spherical ROIs with a radius of 2.5 mm were placed bilaterally at standardized coordinates within the projection (MNI coordination: [116, 110, 99], [64, 110, 99]) and association fiber (MNI coordination: [128, 110, 99], [51, 110, 99]) tracts, as defined by the JHU-ICBM-labels-1 mm atlas. These ROIs were then inversely transformed back into each subject’s native space using the inverse form of the previously computed transformation matrix, with nearest-neighbor interpolation to maintain binary mask integrity. All ROI placements were visually verified and required no manual correction (Figure 1).

The DTI-ALPS index was calculated to assess fluid flow in PVS adjacent to the lateral ventricles. Using the ‘fslstats’ command in FSL, the following values were extracted from the diffusivity maps within the respective ROIs: Mean *x*-axis diffusivity (Dxx) within the projection fiber ROIs (Dxx_proj), Dxx within the association fiber ROIs (Dxx_assoc), Mean *y*-axis diffusivity (Dyy) within the projection fiber ROIs (Dyy_proj), and Mean *z*-axis diffusivity (Dzz) within the association fiber ROIs (Dzz_assoc). The DTI-ALPS index for each hemisphere was computed as follows: DTI-ALPS Index = mean(Dxx_proj, Dxx_assoc)/mean(Dyy_proj, Dzz_assoc) [24].

#### 2.4.2. iALPS Pipeline

All diffusion-weighted data were processed using a fully automated pipeline integrating tools from FSL and AFNI (version 24.3.06, https://github.com/Winniework/Automated-DTI-ALPS-pipeline, accessed on 17 February 2025.). Each subject’s FA map was registered to MNI space as described in Section 2.4.1, and the resulting transformation was applied to the primary eigenvector (V1) and full tensor images with proper reorientation. An FA-weighted RGB colormap was generated from the V1 components, with red, green, and blue channels representing left–right, anterior–posterior, and superior–inferior fiber orientations, respectively.

**Figure 2:**
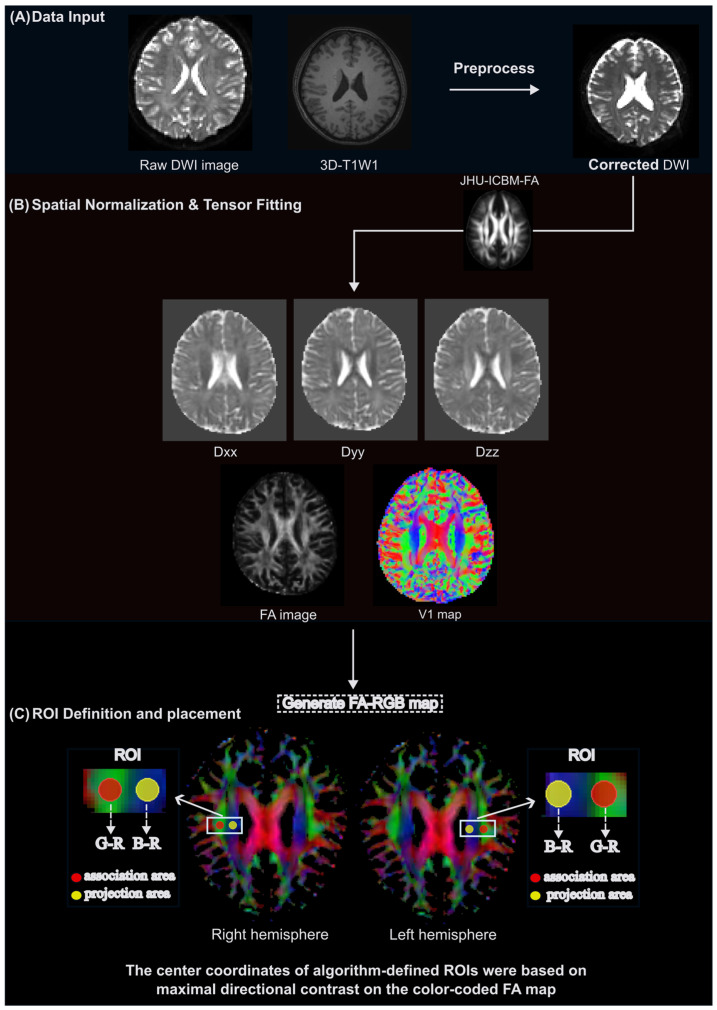
Workflow of the individualized (iALPS) pipeline for DTI-ALPS index computation. The pipeline consists of three major steps. (**A**) Data input and preprocessing: raw diffusion-weighted imaging (DWI) and T1-weighted images were preprocessed using FSL and AFNI, resulting in artifact-corrected diffusion data. (**B**) Tensor fitting and spatial normalization: diffusion tensors were fitted to generate FA, Dxx, Dyy, Dzz, and primary eigenvector (V1) maps. Each subject’s FA map was linearly normalized to the ICBM152 template in MNI space, and the transformation matrix was applied to the V1 and tensor volumes with tensor reorientation. A color-coded FA map was then derived from the V1 components weighted by FA, where the green, blue, and red channels represent anterior–posterior, superior–inferior, and left–right orientations, respectively. Anatomical corridors were defined based on the JHU atlas to constrain the search space. (**C**) ROI definition and placement: within these atlas-defined corridors, optimal ROI locations were identified algorithmically by maximizing directional contrast on the color-coded FA map. Specifically, the association fiber ROI was defined at voxels with the highest (Green-Red) value, and the projection fiber ROI at voxels with the highest (Blue-Red) value. This subject-specific and fully automated procedure minimizes contamination from non-target fibers while maintaining consistency across subjects. The final ROIs were automatically overlaid on each subject’s FA color map for visual verification and quality assurance.

For each hemisphere, optimal ROI locations were determined algorithmically by scanning predefined anatomical corridors. Instead of fixed coordinates, voxel clusters were selected by maximizing diffusion along target axes while minimizing signal along the perivascular direction, yielding direction-contrast maps that enhance orthogonal fiber specificity consistent with the DTI-ALPS theory. Specifically, the centers of ROIs for association and projection fibers were automatically placed at voxels with the highest (G − R) and (B − R) values, respectively. All ROIs were visually verified on orientation-encoded maps (Figure 2). Mean diffusivities (Dxx, Dyy, Dzz) were extracted, and the DTI-ALPS index was calculated as described in Section 2.4.1.

### 2.5. Statistical Analysis

Statistical analyses were performed in Python (v3.0) and R (v4.4.3), and data visualization was conducted in R (v4.3.3). Continuous variables were first assessed for normality using the Kolmogorov–Smirnov test. Normally distributed variables were compared across groups using one-way analysis of variance (ANOVA), while non-normally distributed variables were analyzed using the Kruskal–Wallis test. Categorical variables (e.g., sex) were compared using the chi-square test.

Group differences in the DTI-ALPS index were examined using an inverse probability of treatment weighting (IPTW) approach. Propensity scores for the three groups (BD-D, BD-M, and HCs) were estimated via logistic regression, with age and sex included as covariates. Stabilized weights were derived with truncation at the 99th percentile. Covariate balance was assessed using standardized mean differences (SMDs). After weighting, the groups were balanced with respect to age and sex, allowing for a direct comparison of the DTI-ALPS index across diagnostic groups. Weighted least squares (WLS) regression with robust variance estimation was then applied to compare groups across diagnostic categories. A sensitivity analysis was further conducted with additional adjustment for years of education. Multiple testing correction across three groups was conducted using the false discovery rate (FDR) method.

To evaluate the agreement between the cFSL and iALPS pipelines, Pearson’s correlation coefficient (r) and Spearman’s rank correlation coefficient (ρ) were calculated for each hemisphere to assess linear and monotonic relationships, respectively. Lin’s concordance correlation coefficient (CCC) was used to quantify overall agreement between the two measurement approaches.

## 3. Results

### 3.1. Demographic, Clinical, and Cognitive Characteristics

Demographic and clinical characteristics differed across the three groups (BD-D, BD-M, HC), which are summarized in Table 1. Age did not significantly differ among groups (χ^2^ = 1.135, *p* = 0.567). Significant group differences were observed for years of education (χ^2^ = 20.308, *p* < 0.001), with both BD-D and BD-M having fewer years of schooling than HC (BD-D vs. HC: *p* = 0.0307; BD-M vs. HC: *p* < 0.001). IQ also differed significantly across groups (χ^2^ = 20.655, *p* < 0.001), with both BD-D and BD-M showing lower IQ scores than HC (BD-D vs. HC: *p* = 0.0279; BD-M vs. HC: *p* < 0.001).

Analyses of clinical symptom scales were restricted to patient groups. PANSS total scores were significantly different across groups (χ^2^ = 7.092, *p* = 0.0077), with higher scores in BD-M compared to BD-D (*p* = 0.0077). HAMD scores also differed significantly (F = 85.921, *p* < 0.001), with BD-D showing higher depressive severity than BD-M (*p* < 0.001). YMRS scores showed a strong group effect (χ^2^ = 56.808, *p* < 0.001), with BD-M exhibiting markedly higher manic symptoms than BD-D (*p* < 0.001). Gender distribution did not significantly differ among groups (χ^2^ = 3.659, *p* = 0.1605).

### 3.2. Comparison of the ALPS Index of the Two Pipelines

To assess the agreement between the cFSL and iALPS pipelines in quantifying the DTI-ALPS index, correlation and concordance analyses were performed.

**Figure 3:**
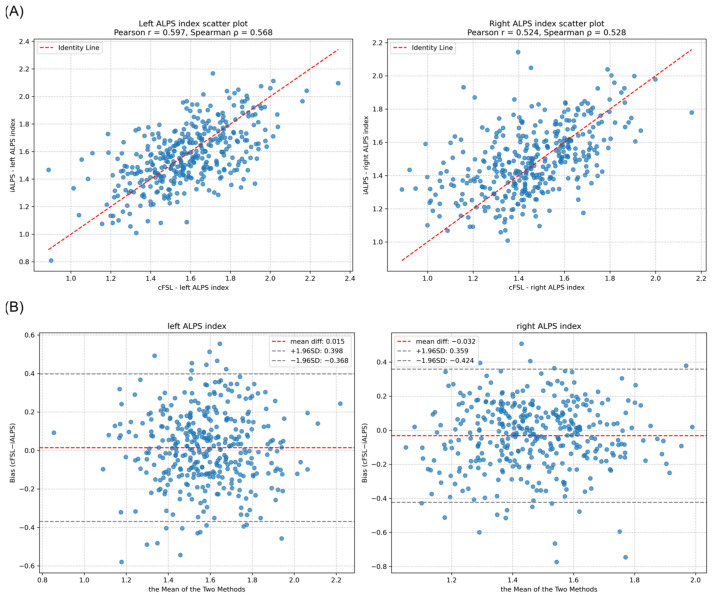
Correlation and concordance analysis of ALPS indices derived from cFSL and iALPS pipelines. (**A**) The dashed line represents the identity line (y = x). A significant positive correlation was observed between the two methods in both hemispheres (**Left**: Pearson’s r = 0.597, *p* < 0.001; **Right**: Pearson’s r = 0.524, *p* < 0.001). (**B**) The central dashed line represents the mean difference (bias) between the two methods. The upper and lower dashed lines represent the 95% limits of agreement (mean difference ± 1.96 × SD of the differences); CCC = 0.60 (**left**)/0.52 (**right**).

For the left hemisphere, moderate concordance between the cFSL and the iALPS pipelines was observed (Pearson’s r = 0.597, *p* < 0.001; Spearman’s ρ = 0.568, *p* < 0.001, and CCC = 0.60). For the right hemisphere, the concordance was slightly lower but remained significant (Pearson’s r = 0.524, *p* < 0.001; Spearman’s ρ = 0.528, *p* < 0.001; CCC = 0.52). The moderate positive bias and wide limits of agreement suggest the presence of a systematic difference and incomplete interchangeability between the two measurement frameworks (Figure 3).

### 3.3. Group Differences in ALPS Index Based on cFSL Pipeline

**Figure 4:**
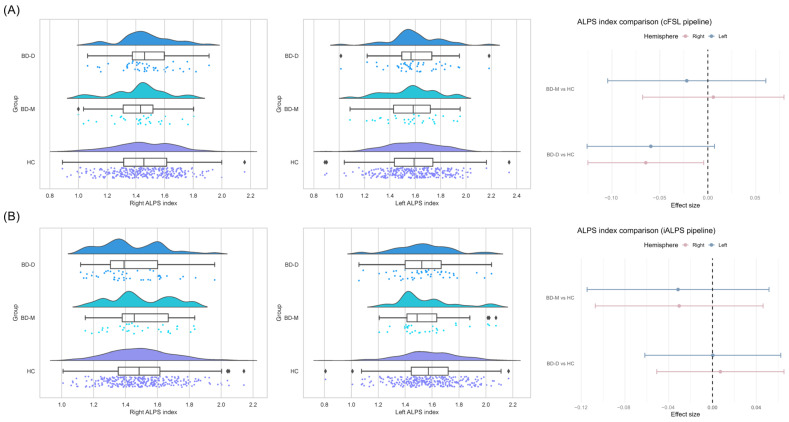
Group differences in DTI-ALPS index derived from the cFSL and iALPS pipelines. (**A**) Group differences in DTI-ALPS index derived from the cFSL pipeline. **Left**: raincloud plot showing the distribution of the right hemispheric ALPS index (Right ALPS index) across diagnostic groups. **Median**: raincloud plot showing the distribution of the left hemispheric ALPS index (Left ALPS index). The raincloud plots combine boxplots, kernel density estimates, and raw data points to visualize the full distribution of the data. **Right**: forest plot summarizing the mean differences (beta coefficients) and 95% confidence intervals in the ALPS index between each patient group and healthy controls, derived from inverse probability of treatment weighting models. Red and blue markers represent left- and right–hemispheric results, respectively. Specific beta coefficients and 95% confidence intervals for each group are as follows: BD-D (*n* = 45): left [−0.062, 0.062], right [−0.051, 0.065]; BD-M (*n* = 32): left [−0.115, 0.052], right [−0.064, 0.022]. All models were adjusted for age and gender. (**B**) Group differences in DTI-ALPS index derived from the iALPS pipeline. **Left**: raincloud plot showing the distribution of the right hemispheric ALPS index (ALPS_R) across diagnostic groups. **Median**: raincloud plot showing the distribution of the left hemispheric ALPS index (ALPS_L). The raincloud plots combine boxplots, kernel density estimates, and raw data points to visualize the full distribution of the data. **Right**: forest plot summarizing the mean differences (beta coefficients) and 95% confidence intervals in the ALPS index between each patient group and healthy controls, derived from inverse probability of treatment weighting models. Red and blue markers represent left- and right-hemispheric results, respectively. Specific beta coefficients and 95% confidence intervals for each group are as follows: BD-D (*n* = 45): left [−0.126, 0.007], right [−0.125, −0.004]; BD-M (*n* = 32): left [−0.104, 0.060], right [−0.068, 0.079]. All models were adjusted for age and gender. In the raincloud plots, green, blue, and purple indicate the BD-D, BD-M, and HC groups, respectively; Diamond symbols indicate outlier data points. In the forest plot, blue indicates patient groups (BD-D and BD-M), and pink indicates the HC group.

After applying inverse probability of treatment weighting (IPTW) for age and sex, covariate balance between each patient group and the HC group was excellent (absolute standardized mean difference < 0.1 for all variables, Table 2). Weighted linear regression analyses showed no significant group differences in the cFSL-derived ALPS index for either hemisphere (Left: *p* > 0.05; Right: *p* > 0.05, uncorrected). Sensitivity analyses additionally adjusting for education years produced parallel null results (Left: *p* > 0.05; Right: *p* > 0.05, uncorrected). Overall, the cFSL pipeline detected no significant glymphatic alterations in the BD-D and BD-M groups relative to the healthy controls (Figure 4).

### 3.4. Group Differences in ALPS Index Based on iALPS Pipeline

Similarly, the automated ROI approach successfully demonstrated covariate balancing through IPTW (Table 3). In the left hemisphere, the iALPS-derived ALPS index indicated a trend-level reduction in the BD-D group compared to healthy controls (β = −0.059, uncorrected *p* = 0.079). However, this effect was not statistically significant after applying FDR correction (FDR-adjusted *p* = 0.318). The BD-M group exhibited no significant differences in the left ALPS index in either the primary or sensitivity analyses (all *p* > 0.05, uncorrected). The iALPS pipeline also revealed a significant reduction in the ALPS index in the right hemisphere for the BD-D group relative to healthy controls (β = −0.065, uncorrected *p* = 0.036). Although this association weakened after FDR correction (FDR-adjusted *p* = 0.071), it represented the largest effect size among all group comparisons. The BD-M group demonstrated no significant alterations in the right ALPS index (uncorrected, *p* > 0.05) (Figure 4). Taken together, while the cFSL pipeline produced uniformly null findings, the iALPS pipeline revealed a right-lateralized reduction in glymphatic function in patients with bipolar depression. This effect remained significant after correction, highlighting the greater sensitivity of the individualized ROI selection approach for detecting subtle variations of the glymphatic function.

## 4. Discussion

The present study produced two principal findings regarding the assessment of glymphatic function in young adults with BD in different stages. First, we observed moderate concordance between the cFSL and iALPS pipelines for computing the ALPS index. Second, while the cFSL pipeline showed no group-level alterations, the iALPS pipeline revealed a trend-level reduction in the ALPS index in BD-D patients, particularly in the right hemisphere. These findings highlight the variability in methodologies for assessing glymphatic function and emphasize that the chosen analytic approach can significantly affect the detection of subtle physiological changes in psychiatric populations.

The difference in methodology between the two pipelines raises an important issue in DTI-ALPS research. Manual ROI placement in the original ALPS method introduces inter-rater variability and poor reproducibility [13,24,36,37]. Even slight errors in ROI placement can significantly compromise the comparability of findings across studies [25,38,39]. Template-based approaches, such as the cFSL pipeline we used [32], mitigate operator bias by registering data to standardized spaces and applying predefined coordinates [40,41], with refinements using template atlases to more accurately delineate the locations of projection and association fibers [38,42,43]. However, it still suffers from imperfect anatomical correspondence between predefined template ROIs and the true PVS [44,45,46]. The iALPS pipeline addresses this by identifying subject-specific ROIs exhibiting PVS-specific diffusivity characteristics, thereby reducing mislocalization and contamination from non-target fibers. In our dataset, moderate cross-method correlations (Pearson ≈ 0.60; Lin’s CCC ≈ 0.55) indicate that the cFSL and iALPS pipelines captured related but non-identical signals. The iALPS pipeline’s ability to detect BD-D alterations likely reflects improved anatomical specificity. The existing literature on the ALPS index in BD remains limited and inconsistent, with one study employing template-derived fixed ROIs [4] and the other using manually delineated ROIs on individual FA maps [5]. This direct comparison demonstrates that methodological rigor critically shapes glymphatic findings in psychiatric diseases, particularly when effects are subtle.

The iALPS pipeline detected a reduced right-hemispheric ALPS index in the young adult cohort with BD-D but not in those with BD-M. This phase- and hemisphere-specific pattern broadens our understanding of the emerging picture of glymphatic function in BD. Two prior studies that yielded discordant ALPS index results both examined chronic BD patients (mean age ≈ 48/35 years) and did not stratify analyses by mood phases [4,5]. Recently, one investigation focusing on first-episode, drug-naïve BD patients (mean age ≈ 20 years) analyzed depressive, manic, and mixed phases separately, finding ALPS index reductions specifically in the mixed phase [47]. Together with our findings in early BD-D, these results preliminarily indicate that glymphatic alterations are present in the early stages of BD. However, the specific mood phase in which they occur remains unclear. Although we did not include a mixed-phase subgroup, the use of the iALPS pipeline and the well-balanced BD-D and BD-M samples enabled a robust comparison between depressive and manic phases.

In addition, our study extends this evidence by demonstrating that reductions in ALPS indices showed a right-hemispheric predominance. This hemispheric pattern aligns with convergent neuroimaging and neurophysiological evidence indicating right-dominant abnormalities in BD-D [48,49], including reduced cortical excitability [50], lower gyrification [51], pronounced reduction in white-matter integrity [52], and impaired suppression of right-hemispheric motor activity [53]. Thus, our findings provide a new insight into this right-lateralized pathophysiology during early BD-D. Moreover, the glymphatic impairment observed here may reflect a specific inflammatory mechanism in BD-D, as supported by previous reports of a negative association between ALPS indices and the free water fraction [5,47], an imaging marker linked to neuroinflammation. Taken together, the ALPS index emerges as a promising biomarker for glymphatic impairment in early-stage BD. However, methodological heterogeneity and cohort differences across existing studies preclude definitive conclusions. Furthermore, while the observed right-hemispheric reduction aligns with prior evidence of right-sided vulnerability in BD-D, it is important to emphasize that this finding emerged at a trend level and requires further confirmation. Future longitudinal and age-stratified studies are needed to delineate the developmental trajectory of glymphatic function and its phase-specific feature in early BD [3,6,54].

A large cohort of young adults strengthens our study, however, several limitations still warrant consideration. First, the cross-sectional design prevents determining whether ALPS index reductions in BD-D represent state effects or enduring traits. Furthermore, residual confounding is possible despite IPTW adjustment, particularly given group differences in education, cognitive level, and the lack of objective sleep assessments, a known modulator of glymphatic flow [55]. Multi-site replication studies with diverse cohorts are needed to confirm the robustness and external validity of our observations. Finally, the ALPS index remains an indirect proxy influenced by neurovascular coupling and vascular/perivascular anatomy [25]. Integration with direct glymphatic imaging and physiological markers is required to validate its biological specificity in the future [56,57]. This study demonstrates that the choice of ROI placement strategy critically affects DTI-ALPS measurements, showing only moderate agreement between the innovative iALPS pipeline and the cFSL pipeline. By applying the iALPS pipeline, we revealed a trend-level phase-specific reduction in ALPS indices in young patients with BD during depressive episodes. In contrast, no significant alterations were detected in BD-M. These findings suggest that glymphatic dysfunction in psychiatric disorders may vary with disease stage, emphasizing the methodological importance of the iALPS pipeline for detecting subtle, state-related alterations in glymphatic function. Future large-scale, longitudinal, and multimodal investigations are warranted to elucidate the potential role of glymphatic function in the pathophysiology and clinical progression of major psychiatric disorders.

## Figures and Tables

**Table 1:** Demographic and clinical characteristics.

Characteristic	BD-D (*n* = 45)	BD-M (*n* = 32)	HC (*n* = 289)	*p*-Value
Demographics				
Age (years)	25.29 ± 5.98	24.41 ± 6.31	25.49 ± 5.71	>0.05
Sex, male, *n* (%) (male/female)	22 (48.9)	11 (34.4)	99 (34.3)	>0.05
Education (years)	14.38 ± 2.71	13.50 ± 3.12	15.66 ± 2.41	<0.001
WAIS	111.16 ± 12.69	107.25 ± 11.70	116.15 ± 12.27	<0.001
Clinical measures				
PANSS	52.98 ± 10.50 (*n* = 45)	66.28 ± 26.53 (*n* = 32)	NA	<0.05
HAMD	15.13 ± 4.46 (*n* = 45)	6.09 ± 3.85 (*n* = 32)	NA	<0.001
YMRS	1.44 ± 1.79 (*n* = 45)	18.50 ± 6.15 (*n* = 32)	NA	<0.001

Data are presented as mean ± standard deviation for continuous variables and percentage (%) for categorical variables. Sample sizes for clinical measures are indicated in parentheses when they differ from the group totals. *p*-values were derived from the Kruskal–Wallis test for continuous variables and the chi-square test for categorical variables. Post hoc pairwise comparisons with Bonferroni correction were performed for significant findings (all *p* < 0.05). BD-D: bipolar disorder, depressive episode; BD-M: bipolar disorder, manic episode; HC: healthy controls; WAIS: Wechsler Adult Intelligence Scale; PANSS: Positive and Negative Syndrome Scale; HAMD: Hamilton Depression Rating Scale; YMRS: Young Mania Rating Scale; NA: not applicable.

**Table 2:** Between-group comparisons of DTI-ALPS index derived from the cFSL pipeline using inverse probability of treatment weighting: BD-D vs. HC and BD-M vs. HC.

Group	Age (SMD)	Gender (SMD)	Coefficient			*p*-Value		CI_Lower		CI_Upper	
				Left	Right	Left	Right	Left	Right	Left	Right
BD-D (*n* = 45)	0.004	0.030	Main (age + gender)	0.000	0.007	0.993	0.808	−0.062	−0.051	0.062	0.065
			Sensitivity (edu_years)	0.006	0.022	0.845	0.473	−0.059	−0.039	0.072	0.084
BD-M (*n* = 32)	0.024	0.010	Main (age + gender)	−0.031	−0.030	0.458	0.437	−0.115	−0.064	0.052	0.022
			Sensitivity (edu_years)	−0.020	−0.008	0.659	0.850	−0.110	−0.042	0.069	0.047

Results were derived from weighted least squares regression models with robust variance estimation, comparing each patient group with healthy controls. For each diagnostic group, two models are presented: the main model adjusted for age and gender, and a sensitivity model additionally adjusted for years of education. Coefficient estimates: corresponding *p*-values and 95% confidence intervals are shown separately for the left and right hemispheres. All statistical tests were two-sided. SMD: standardized mean difference (reflecting covariate balance after weighting, with values < 0.1 indicating good balance). CI_lower: lower bound of 95% confidence interval; CI_upper: upper bound of 95% confidence interval.

**Table 3:** Between-group comparisons of DTI-ALPS index derived from the iALPS pipeline using inverse probability of treatment weighting: BD-D vs. HC and BD-M vs. HC.

Group	Age (SMD)	Gender (SMD)	Coefficient			*p*-Value		CI_Lower		CI_Upper	
				Left	Right	Left	Right	Left	Right	Left	Right
BD-D (*n* = 45)	0.004	0.030	Main (age + gender)	−0.059	−0.065	0.079	0.036	−0.126	−0.125	0.007	−0.004
			Sensitivity (edu_years)	−0.050	−0.053	0.157	0.094	−0.120	−0.115	0.019	0.009
BD-M (*n* = 32)	0.024	0.010	Main (age + gender)	−0.022	0.006	0.601	0.880	−0.104	−0.068	0.060	0.079
			Sensitivity (edu_years)	−0.016	0.0156	0.726	0.694	−0.103	−0.061	0.071	0.092

Results from weighted least squares regression models with robust variance estimation, comparing each patient group against healthy controls. Analyses were based on DTI-ALPS indices generated through the individualized DTI-ALPS pipeline. For each diagnostic group, two models are presented: the main model (adjusted for age and gender) and a sensitivity model (additionally adjusted for years of education). Coefficient estimates, *p*-values, and 95% confidence intervals are shown separately for left and right hemispheric analyses. All statistical tests were two-sided.

## Data Availability

Imaging data supporting the findings of this study are available from the corresponding author upon reasonable request. All codes for analysis can be downloaded online from following website: for iALPS pipeline (https://github.com/Winniework/Automated-DTI-ALPS-pipeline, accessed on 17 February 2025).

## Supplementary Materials

The following supporting information can be downloaded at: https://www.mdpi.com/article/10.3390/biomedicines14030699/s1, Table S1: Medication situation data of in BD patients.

## Abbreviations

The following abbreviations are used in this manuscript:

BD	Bipolar Disorder
DTI-ALPS	Diffusion Tensor Image Analysis along the Perivascular Space
cFSL	Conventional FSL-based (pipeline)
iALPS	Individualized ALPS (pipeline)
HC	Healthy Control(s)
CNS	Central Nervous System
CSF	Cerebrospinal Fluid
ISF	Interstitial Fluid
PVSs	Perivascular Spaces
AQP-4	Aquaporin-4
SCZ	Schizophrenia
MDD	Major Depressive Disorder
BD-M	Bipolar disorder during a manic episode
BD-D	Bipolar disorder during a depressive episode
SCID-4-P	Structured Clinical Interview for DSM-IV Disorders, Patient Version
DSM-IV	Diagnostic and Statistical Manual of Mental Disorders, Fourth Edition
SCID-4-NP	Structured Clinical Interview for DSM-IV Disorders, Non-patient Version
HAMD	Hamilton Depression Rating Scale
YMRS	Young Mania Rating Scale
MRI	Magnetic Resonance Imaging
T1w	T1-Weighted
MPRAGE	Magnetization-prepared rapid gradient-echo
TR	Repetition Time
TE	Echo Time
TI	Inversion Time
EPI	Echo-Planar Image
FOV	Field of View
FA	Fractional Anisotropy
ICBM	International Consortium for Brain Mapping
IPTW	Inverse probability of treatment weighting
WLS	Weighted Least Squares
FDR	False Discovery Rate
CCC	Lin’s Concordance Correlation Coefficient
