# Supplementary material for: Individualized DTI-ALPS Identifies Phase-Specific Glymphatic Dysfunction in Early-Stage Bipolar Disorder
Source: Biomedicines. 2026 Mar 17;14(3):699. doi: 10.3390/biomedicines14030699 (PMC13024597; doi:10.3390/biomedicines14030699)
Supplement: Supplementary file 1 [file biomedicines-14-00699-s001.zip › biomedicines-4167767-supplementary.pdf]

Supplementary Table S1. Medication situations data of in BD patients

Counts of patients in different medication situations	BD-D (n=45)[1]	BD-M (n=32)[2]
Non-medication	11	15
Medication	34	17
--Mood stabilizers (MS)[3]	2	0
--Antipsychotics (AP)[4]	2	2
--Antidepressants (AD)[5]	5	2
--MS+AP	5	7
--MS+AD	9	1
--AP+AD	2	0
--MS+AP+AD	9	5
With other medications[6]	11	7

1 bipolar disorder patients in a depressive state

2 bipolar disorder patients in a manic state

3 mood stabilizers include lithium, valproate, and lamotrigine.

4 antipsychotics include olanzapine, clozapine, quetiapine, risperidone, paliperidone, aripiprazole, ziprasidone, blonanserin, and haloperidol.

5 antidepressants include fluoxetine, sertraline, duloxetine, paroxetine, venlafaxine, mirtazapine, escitalopram, citalopram hydrobromide, amitriptyline, and bupropion.

6 other medications include buspirone, tandospirone, lorazepam, alprazolam, estazolam, clonazepam, benzhexol, propranolol, zolpidem tartrate, eszopiclone, and traditional Chinese herb.
